# Twist and miR-34a Are Involved in the Generation of Tumor-Educated Myeloid-Derived Suppressor Cells

**DOI:** 10.3390/ijms141020459

**Published:** 2013-10-14

**Authors:** Xin Wang, Xusheng Chang, Guangzuan Zhuo, Mingjuan Sun, Kai Yin

**Affiliations:** 1 Department of General Surgery, Changhai Hospital, the Second Military Medical University, Shanghai 200433, China; E-Mail: wang_x306@126.com; 2 Department of Gynaecology and Obstetrics, the 306 Hospital of PLA, Beijing 100037, China; 3 Department of General Surgery, Yancheng City First People’s Hospital, Yancheng City 224000, Jiangsu, China; E-Mail: changxush163@163.com; 4 Department of Colorectal Surgery, the Second Artillery General Hospital of PLA, Beijing 10008, China; E-Mail: zhuoguangzan163@163.com; 5 Department of Biochemistry and Molecular Biology, Second Military Medical University, Shanghai 200433, China

**Keywords:** myeloid-derived suppressor cells, twist, miR-34a, dendritic cells, tumor escape

## Abstract

Tumors can induce the generation and accumulation of immunosuppressive cells such as myeloid-derived suppressor cells in the tumor microenvironment, contributing to tumor immunological escapes. Many studies have demonstrated that multiple factors could induce myeloid precursor cells into myeloid-derived suppressor cells, not dendritic cells. In our study, we found that tumor supernatants could induce the generation of myeloid-derived suppressor cells by disturbing the development of dendritic cells. Twist and miR-34a may regulate the effect of tumor cells inducing myeloid-derived suppressor cells via TGF-β and/or IL-10.

## 1. Introduction

The tumor microenvironment is well known to be immunosuppressive [1–4]. Tumor-derived factors such as TGF-β, VEGF, IL-10, and PGE_2_ and other factors such as gangliosides and lactate have been verified to be able to regulate differentiation and function of dendritic cells (DC) and T cells [5–7]. It has been well established that the tumor microenvironment could drive imDC (immature dendritic cells) to differentiate into CD11b^high^Ia^low^ regulatory DC via TGF-β and PGE_2_, and that regulatory DC suppress T cells response [8]. Interestingly, the development of DC could be disturbed in the early period. Myeloid precursor cells may differentiate into DC, macrophages, granulocytes and mast cells depending on the microenvironmental conditions. They might also stay in non-differentiated mixed form, which include myeloid-derived suppressor cells (MDSC) [9,10].

MDSC and DC share the same myeloid precursor cell, and play very different roles in the immune response. MDSC represent a mixed population of immature myeloid cells including DC precursors, and were firstly found in tumor tissues and in the lymph nodes of tumor-bearing mice [11]. It is known that proinflammatory cytokines, such as IL-1β, IL-6 and bioactive lipid PGE_2_ could induce the accumulation of MDSC.

MDSC can suppress the immune response via arginase, iNOS [12,13], ROS [14–21], and Foxp3^+^ regulatory cells [22,23]. DC showed a capacity to initiate innate and adaptive response [24–27] and DC can induce T cell immune response by presenting the foreign antigen, upregulating co-stimulatory molecules and releasing proinflammatory cytokines. And in the tumor microenvironment, regulatory DC suppress T cells function via arginase I [8]. It should be noted that regulatory DC (regDC) are very different from conventional DC, and regDC derived from different organ stromal microenvironments are generated through different mechanisms.

Twist is a transcription factor that belongs to the bHLH family and expressed in and associated with many types of aggressive tumors, including breast cancer [28], hepatocellular carcinoma [29,30], prostate cancer [31,32], oesophageal squamous cell carcinoma [33,34], bladder cancer [35,36] and pancreatic cancer [37]. Twist plays multiple roles in cancer initiation, progression and metastasis [38]. More specifically, Twist can override oncogene-induced cell senescence and apoptosis, increase cancer cell resistance to chemotherapy, enhance cancer stem cell (CSC) population, and facilitate cancer cell invasion and metastasis [38]. So, we guessed that Twist may increase the population of MDSC or enhance the capacity of MDSC.

MicroRNA (miRNAs) are a conserved class of non-coding 20–22 nt small RNAs that regulate gene expression by binding to mRNA, leading to mRNA degradation or inhibition [39]. And a global decrease in microRNA (miRNA) levels is often observed in human cancer [40], indicating that small RNAs may have an intrinsic function in tumor suppression. MicroRNA miR-34 was fully studied and was identified as a p53 target and a potential tumor suppressor [40–43]; miR-34 may also act in concert with other effectors to inhibit inappropriate cell proliferation.

In our research, we tried to test whether tumor supernatant could induce myeloid precursor cells into MDSC, not DC, and elucidated the roles of Twist and miR-34a in the process. We hope that this research may provide a new understanding of the mechanism of tumor immune escape.

## 2. Results and Discussion

### 2.1. Tumor Culture Supernatants Disturb the Development of the Bone Marrow-Derived Dendritic Cell

Bone marrow-derived dendritic cells were produced by standard protocol *in vitro*, and the process of transduction from myeloid precursors into DC needs 5 or 6 days [44–46]. Myeloid precursor cells washed from mice bone marrow were co-cultured with NIH3T3, Hepa1-6, and CT26 cell lines supernatants as the method indicated.

**Figure 1:**
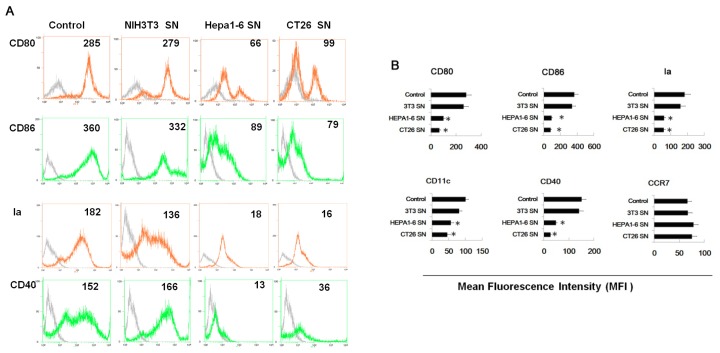

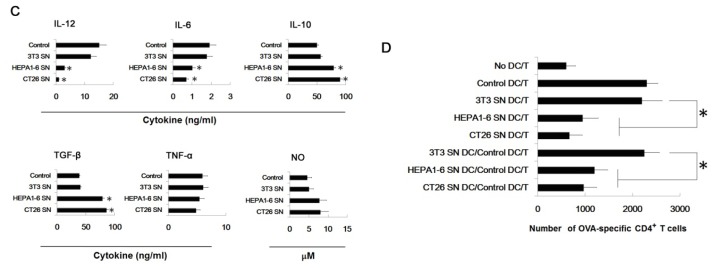
Tumor supernatants pretreated cells showed an altered phenotype and cytokine profile, and a reduced ability to stimulate proliferation of T cells. Myeloid precursor cells were generated from mice (BALB/c mice, 5–6 weeks of age) femur bone marrow suspensions by depletion of red cells. These cells were cultured in 24 well plates using 1 × 10^6^ cell/well. All groups were cultured with GM-CSF, IL-4 during the entire process. 24 h tumor or NIH3T3 supernatants were collected and treated with myeloid precursor cells for 5 days. SN, supernatants (**A**) Cell pretreated with tumor or NIH3T3 supernatants were labeled with antibody to CD80, CD86, Ia, and CD40, respectively, for phenotypic analysis by flow cytometry. Dotted lines represent isotype control. Number in histograms indicated geometric mean fluorescence intensity; (**B**) Mean fluorescence intensity of CD80, CD86, Ia, CD40, CD11c and CCR7. Results were the mean ± SD of three independent experiments. * *p* < 0.05; (**C**) NO expression and cytokine profile of cells from different pretreated groups for 24 h. On day 6, cells of different groups were collected and washed with PBS 3 times and placed in wells for another 24 h. Then IL-12, IL-6, TNF, IL-10, TGF-β were assayed by ELISA and NO were assayed by Griess. Results were the mean ± SD of triplicate wells. * *p* < 0.05; NS, no significance; (**D**) CD4^+^ T cells from DO11.10 OVA323–339 specific mice were co-cultured with cells from all groups, 5 days later, the total number of viable CD4^+^ T cells (CD4^+^ 7AAD^−^) cells in each well was measured by flow cytometry. Results are the mean ± SD of three independent analyses. * *p* < 0.05.

As shown in Figure 1A, after co-culture with supernatants of fibroblast (NIH3T3) or tumor cells (Hepa1-6, CT26), these cells showed a very different phenotype. Cells co-cultured with tumor supernatant showed a lower expression of CD80, CD86, Ia, CD11c and CD40, and the difference of CCR7 among these three groups was not significant (CD11c is generally expressed by dendritic cells; Ia belongs to Class II MHC antigen; CD40, CD80, CD86 are co-stimulatory molecules of dendritic cells; CCR7 is a chemotactic receptor that induces the dendritic cell to the lymphatic system.). Tumor supernatant treated cells secreted lower levels of IL-12 and IL-6, higher levels of IL-10, TGF-β and NO.

Wen next tested whether these cells exerted immunoregulatory function by stimulating antigen-specific T cells responses. We found that tumor supernatant treated cells showed reduced ability to stimulate proliferation of OVA-specific CD4^+^ T cells. Significantly, we added cells co-cultured with supernatants of tumor cells into the mDCs/CD4^+^ T co-culture system; we found the T cells proliferation *in vitro* was partly suppressed.

### 2.2. Supernatants of Tumor Cells Inhibit the DC Differentiation from Myeloid Precursor Cells, and Promote MDSC Accumulation

**Figure 2:**
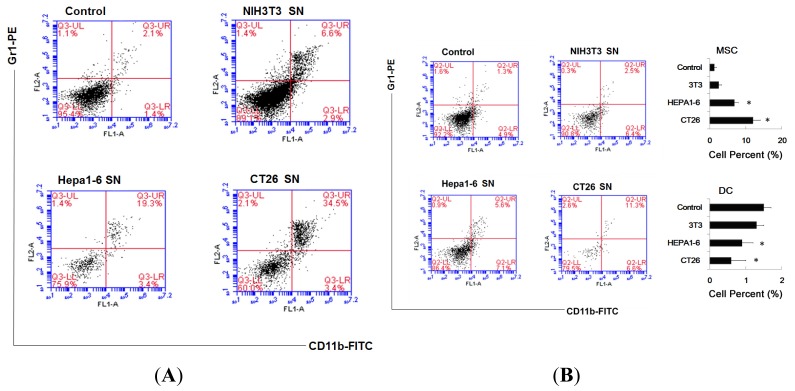
Supernatants of tumor cells inhibit DC and promoted MDSC accumulation. (**A**) Myeloid precursor cells co-cultured with supernatants method was indicated as previously. After co-culture, treated cells were washed with PBS 3 times, and doubled-labeled with Ab to Gr1 and CD11b, for phenotypic analysis by flow cytometry; (**B**) The percent of MDSC and DC in the spleen of tumor-bearing mice were assayed by FACS. Spleens were isolated from tumor-bearing mice, after depletion of red blood cells, were labeled Ab to Gr1 and CD11b or labeled Ab to CD11c. Percent of MDSC (Gr1^+^CD11b^+^) and percent of DC(CD11c^+^) were calculated by FACS. * *p* < 0.05.

The cells treated with supernatants of tumor cells showed low expression of co-stimulatory molecules and a cytokine profile of low expression of IL-12, IL-6 and high expression IL-10 and TGF-β. In mice, phenotype of MDSC is Gr1^+^CD11b^+^[11], so we analyzed these population by double staining, and found more double-positive cells (Gr1^+^CD11b^+^ cells) existed in Hepa1-6 cells group (19.3%) and CT26 cells group (34.5%) (Figure 2A). To exclude the possibility that this is the artificial phenomenon observed *in vitro*, we tried to confirm whether Gr1^+^CD11b^+^ cells exist in spleen of tumor-bearing mice. As shown in Figure 2B, Gr1^+^CD11b^+^ cells did exist in the spleen and constituted 11.3% of total karyocyte in spleen; in addition the percent of DC (CD11c^+^ cells) in spleen of tumor-bearing mice is lower than negative control mice.

### 2.3. Twist Regulated the Effect of Tumor Cells of Inducing Myeloid Precursor Cells into MDSC

**Figure 3:**
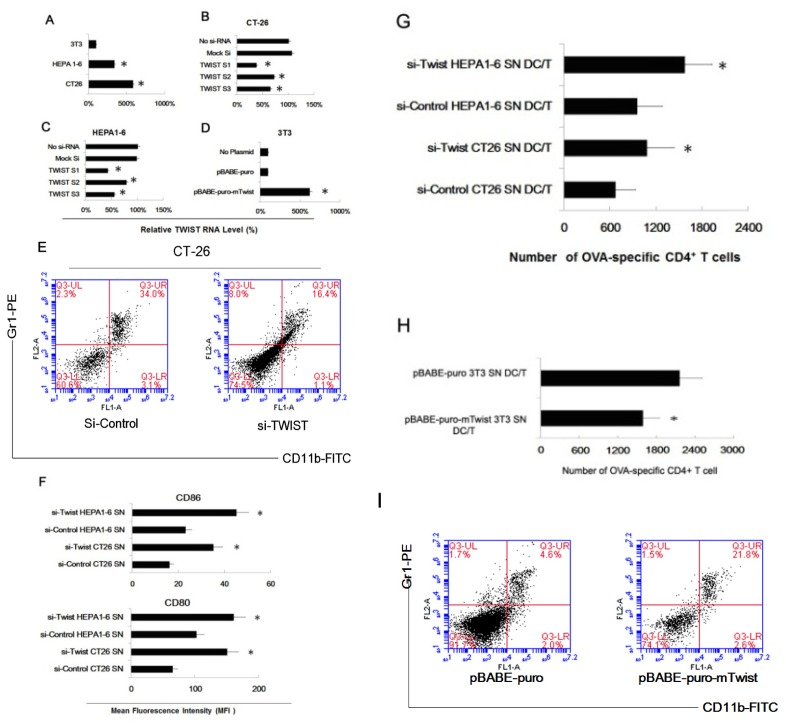
Twist regulated the effect of tumor cells of inducing myeloid precursor cells into MDSC. (**A**) Firstly, the relative mRNA expression of Twist of CT26, Hepa1-6 and NIH3T3 cells were assayed by real time PCR. Histograms represent the relative mRNA expression. Results were the mean ± SD of three independent assays. * *p* < 0.05; (**B**) CT26 cells were transfected with Twist siRNA (Twist S1, Twist S2, Twist S3 and Mock siRNA) as method indicated. Relative Twist mRNA expression was assayed by real time PCR. Results were the mean ± SD of three independent assays. * *p* < 0.05; (**C**) Hepa1-6 cells were transfected with Twist siRNA (Twist S1, Twist S2, Twist S3 and Mock siRNA) as method indicated. Relative Twist mRNA expression was assayed by real time PCR. Results were the mean ± SD of three independent assays. * *p* < 0.05; (**D**) NIH3T3 were transfected with Twist overexpression plasmid (pBABE-puro-mTwist), negative control plasmid (pBABE-puro) and blank control (No Plasmid). The relative Twist mRNA expression was then assayed by real time PCR. Results were the mean ± SD of three independent assays. * *p* < 0.05; (**E**) The supernatants of siRNA transfected CT26 cells were collected and co-cultured with myeloid precursor cells, and then these cells were purified for MDSC phenotype identification. The percent of Gr1^+^CD11b^+^ were 16.4% (Twist siRNA) and 34.0% (control siRNA) respectively; (**F**) After co-cultured with supernatants, these cells were labeled with Ab to CD80 and CD86, and assayed by FACS respectively. Results were the mean ± SD of three independent assays. * *p* < 0.05; (**G**) CD4^+^ T cells from DO11.10 OVA323–339-specific (TCR-transgenic × C57BL/6) F1 hybrid mice were co-cultured with pretreated myeloid precursor cells, 5 days later, the total number of viable CD4^+^ T cells (CD4^+^ 7AAD^−^) cells in each well was measured by flow cytometry. Results are the mean ± SD of three independent analysis. * *p* < 0.05 SN, supernatants; (**H**) Twist expression of NIH3T3 cells was upregulated by transfection pBABE-puro-mTwist, then the supernatants of transfected NIH3T3 were collected for co-culture of myeloid precursor cells, and these cells were washed with PBS 3 times and co-cultured with CD4^+^ T cells as previous method indicate. Results are the mean ± SD of three independent analyses. * *p* < 0.05 SN, supernatants; (**I**) After co-cultured with supernatants of pBABE-puro-mTwist transfected NIH3T3, the myeloid precursor cells were labeled with Ab to Gr1 and CD11b, and percent of MDSC (Gr1^+^CD11b^+^) were counted by FACS. The percent of MDSC were 21.8% (pBABE-puro-mTwist) and 4.6% (negative control, pBABE-puro) respectively.

Twist, a master regulator of embryonic morphogenesis, plays an essential role in tumor metastasis. The suppression of Twist expression in highly metastatic mammary carcinoma cell specifically inhibits their ability to metastasize from the mammary gland to the lung [28]. So, we tested the Twist expression of the three cell lines, and found that the expression of Twist in Hepa1-6 and CT-26 cells is higher than in NIH3T3 cells (Figure 3A). Next, we downregulated Twist expression in two tumor cell lines by Twist-siRNA and upregulated the Twist expression by plasmid transfection (pBABE-puro-mTwist). As shown in Figure 3B,C, Twist expression in the two tumor cell line were downregulated by all three Twist siRNA, and Twist siRNA1 showed the most potent ability. After Twist overexpression plasmid (pBABE-puro-mTwist) transfection, the expression of Twist of NIH3T3 was upregulated to a very high level (Figure 3D). Next, we repeated our previous myeloid precursor cells and tumor supernatants co-culture experiments by using the three pretreated cell lines. Similarly, we purified and analyzed tumor supernatants treated myeloid precursor cells, and found a similar phenomenon in both Hepa1-6 and CT-26 supernatant pretreated group. We proved that Twist siRNA could decrease the percent of Gr1^+^CD11b^+^ cells in a population (Figure 3E). And unsurprisingly, CD86 and CD80 expression is higher in Twist-siRNA treated cells than in control (Figure 3F). Twist-siRNA partially restored the ability of the tumor supernatant pretreated cells to stimulate proliferation of OVA-specific CD4^+^ T cells (Figure 3G). Similarly, we upregulated Twist expression of NIH3T3 by transfection pBABE-puro-mTwist, and used supernatants of transfected NIH3T3 cells to co-culture with myeloid precursor cells. We found that the percent of Gr1^+^CD11b^+^ cells in population was increased (Figure 3H) and these cells showed a decreased ability to stimulate proliferation of OVA-specific CD4^+^ T cells (Figure 3I).

### 2.4. MiR-34a Regulated the Effect of Tumor Cells of Inducing Myeloid Precursor Cells into MDSC

**Figure 4:**
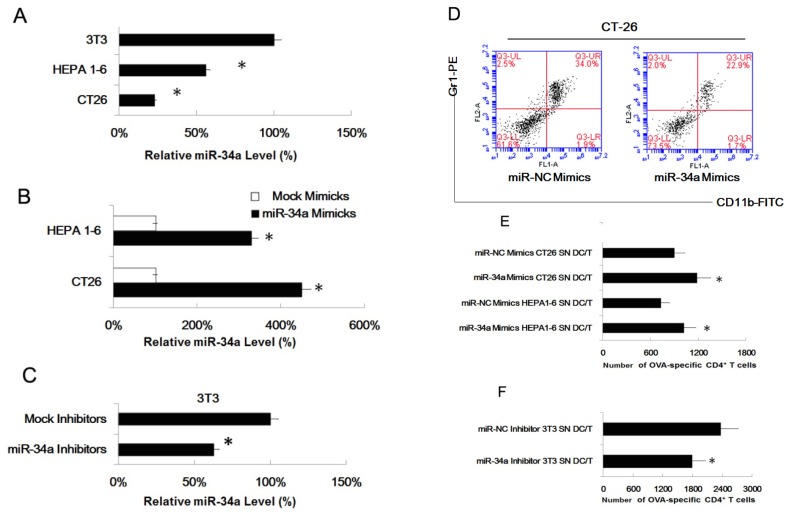
The role of miR-34a in the process of inducing myeloid precursor cells into MDSC. The miR-34a expressions of CT26, Hepa1-6 and NIH3T3 cells were upregulated or downregulated by transfection of miR-34a mimics or specific inhibitors. Supernatants were collected separately following co-cultured experiments with myeloid precursor cells. These cells co-cultured with pretreated supernatants showed a lower percent MDSC phenotype and an increased capacity to stimulate the proliferation of T cells. (**A**) The relative miR-34a expression of CT26, Hepa1-6 and NIH3T3 cells were assayed by real time PCR. Histograms represent the relative mRNA expression. Results were the mean ± SD of three independent assays. * *p* < 0.05; (**B**) After the transfection of miR-34a mimic, the expression of miR-34a of CD26 and Hepa1-6 cells were assayed. The relative expression is shown. Results were the mean ± SD of three independent assays. * *p* < 0.05; (**C**) After the transfection of the miR-34a specific inhibitor, the expression of miR-34a of NIH3T3 cells were assayed. Results were the mean ± SD of three independent assays. * *p* < 0.05; (**D**) miR-34a mimics were transfected into CT26 cell, and the supernatants of transfected CT26 cells were collected and co-cultured with myeloid precursor cells like before. As previously, the percent of MDSC were assayed by FACS: 34.0% (miR-NC mimic) *versus* 22.9% (miR-34a mimic). NS, negative control (**E**) CD4^+^ T cells from DO11.10 OVA323–339-specific (TCR-transgenic × C57BL/6) F1 hybrid mice were co-cultured with pretreated myeloid precursor cells (tumor cell supernatants pretreated), 5 days later, the total number of viable CD4^+^ T cells (CD4^+^ 7AAD^−^) cells in each well was measured by flow cytometry. Results are the mean ± SD of three independent analyses. * *p* < 0.05 SN, supernatants (**F**) CD4^+^ T cells from DO11.10 OVA323–339-specific (TCR-transgenic × C57BL/6) F1 hybrid mice were co-cultured with pretreated myeloid precursor cells (NIH3T3 supernatants pretreated), 5 days later, the total number of viable CD4^+^ T cells (CD4^+^ 7AAD^−^) cells in each well was measured by flow cytometry. Results are the mean ± SD of three independent analyses. * *p* < 0.05 SN, supernatants.

Gene encoding miRNAs in the miR-34 family are direct transcriptional targets of p53, whose induction by DNA damage and oncogenic stress depends on p53 both *in vitro* and *in vivo*. It has been proven the miR-34a is a tumor suppressor, and miR-34a could reduce cell proliferation and invasiveness [40]. Here, we found that the expression of miR-34a is lower in CT26 and Hepa1-6 cells than in NIH3T3 cells (Figure 4A). So, we upregulated the miR-34a expression by miR-34a specific mimics in tumor cell lines (CT-26 and Hepa1-6) and downregulated miR-34a expression in NIH3T3 cells by an miR-34a specific inhibitor (Figure 4B,C). Since the miR-34a expression of CT26 was changed more dramatically than Hepa1-6, we upregulated the miR-34a expression of CT26 cells and found higher expression of miR-34a led a lower percent of Gr1^+^CD11b^+^ cells (22.9%) in the population as comparing with control (34.0%) (Figure 4D). Then the T cell proliferation experiment indicated that higher expression of miR-34a in CT26 resulted in more proliferation of OVA-specific CD4^+^ T cells (Figure 4C) (Figure 4E), and lower expression of miR-34a in NIH3T3 resulted in less proliferation OVA-specific CD4^+^ T cells (Figure 4F).

### 2.5. The Combined Effect of Twist and miR-34a on Inducing MDSC

**Figure 5:**
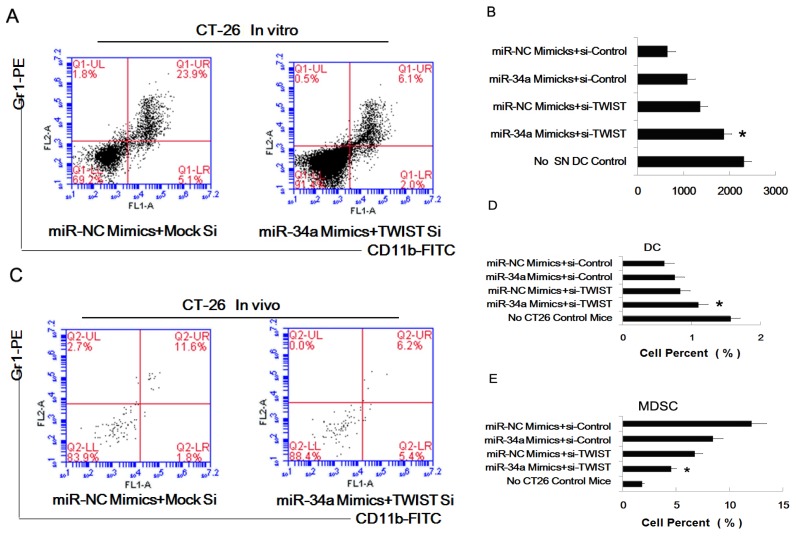
The combined effect of Twist and miR-34a in inducing MDSC. miRNA-34a mimics and Twist siRNA were combined to pretreated tumor cells. The supernatants of pretreated cells were collected and co-cultured with myeloid precursor cells; or the combined treated tumor cells were inoculated s.c. into C57BL/6 mice for the construction of tumor bearing mice model. After co-culture, these cell were purified to assess the percent of MDSC and the capacity to stimulate the proliferation of T cells. (**A**) Percent of MDSC (G1^+^CD11b^+^) in the pretreated cells population was assayed by FACS; (**B**) CD4^+^ T cells from DO11.10 OVA323–339-specific (TCR-transgenic × C57BL/6) F1 hybrid mice were co-cultured with four different pretreated myeloid precursor cells, 5 days later, the total number of viable CD4^+^ T cells (CD4^+^ 7AAD^−^) cells in each well was measured by flow cytometry. Results are the mean ± SD of three independent analyses. * *p* < 0.05 SN, supernatants; (**C**) The spleens of tumor bearing mice were isolated for assaying the percent of MDSC (Gr1^+^CD11b^+^); (**D**) The percent of MDSC (Gr1^+^CD11b^+^) in spleens of tumor bearing mice were assayed by FACS. There are four different pretreatment combinations. Results are the mean ± SD of three independent analyses. * *p* < 0.05; (**E**) The percent of DC (CD11c^+^) in spleens of tumor bearing mice was assayed by FACS. Results are the mean ± SD of three independent analyses. * *p* < 0.05.

Having shown that both Twist and miR-34a play important roles in the process of inducing marrow precursor cells into MDSC, we decided to investigate treatment with combined Twist knockout and miR-34a mimics. We analyzed the phenotype of the treated myeloid precursor cells and found that combined treatment resulted in a lower percent of Gr1^+^CD11b^+^ cells (6.1% *versus* 23.9%) (Figure 5A). Next we found these pretreated cells showed the most potent ability of stimulating proliferation of OVA-specific CD4^+^ T cells (Figure 5B). And then we transfected CT26 cells with miR-34a mimics and Twist-siRNA, and inoculated these cells s.c. (subcutaneously) into C57BL/6 mice. Two weeks later, spleens of the tumor bearing mice were isolated for assaying the percent of MDSC or DC; a similar phenomenon was seen *in vivo* (6.2% *versus* 11.6%) (Figure 5C). Different combination of transfection pretreatment indicated that combination of miR-34a mimics and Twist siRNA showed the greatest effect (Figure 5D,E).

### 2.6. Tumor-Derived IL-10 and TGF-β Are Responsible for the Differentiation of MDSC

**Figure 6:**
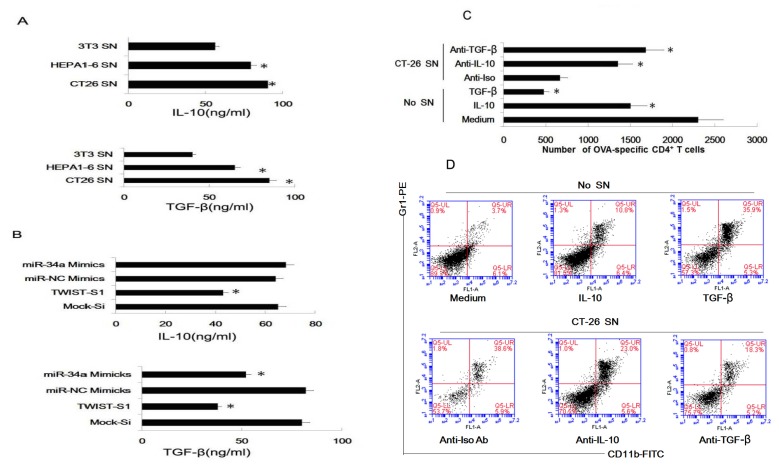
Tumor supernatants induced myeloid precursor cells into MDSC, not DC via TGF-β or IL-10. (**A**) The IL-10 and TGF-β level in supernatant of the three cell lines were assayed by ELISA. Results are the mean ± SD of three triplicate wells. * *p* < 0.05 SN, supernatants; (**B**) The IL-10 and TGF-β level in supernatant of the transfected cells were assayed by ELISA. After plasmid or siRNA transfection, cells were washed by PBS 3 time, and then 24 h supernatants were collected for ELISA assay. Results are the mean ± SD of three triplicate wells. * *p* < 0.05 SN, supernatant; (**C**) CD4^+^ T cells from DO11.10 OVA323–339-specific (TCR-transgenic × C57BL/6) F1 hybrid mice were co-cultured with pretreated myeloid precursor cells, 5 days later, the total number of viable CD4^+^ T cells (CD4^+^ 7AAD^−^) in each well was measured by flow cytometry. Results are the mean ± SD of three independent analyses. * *p* < 0.05 SN, supernatants; (**D**) Percent of MDSC (Gr^+^CD11b^+^) in the pretreated cells population were analyzed by FACS.

To elucidate the mechanism involved in tumor supernatants inducing myeloid precursor cells into MDSC, we assayed cytokines in the supernatants of NIH3T3, Hepa1-6 and CT-26 cells, and found tumor cell lines secreted high level of IL-10 and TGF-β than NIH3T3 (Figure 6A). We also proved that miR-34a mimics and Twist siRNA could suppress the secretion of IL-10 and TGF-β. It is noted that Twist siRNA could inhibit the secretion of both IL-10 and TGF-β, and miR-34a could only suppress the secretion of TGF-β (Figure 6B). At last, we found that recombinant TGF-β and IL-10 showed a similar effect comparing with tumor supernatants, and neutralizing Abs to TGF-β and IL-10 could reduce the effect of tumor supernatants. As Figure 6C,D indicated, recombinant TGF-β and IL-10 could lead to the accumulation of MDSC, and reduce the capacity of co-cultured cells to stimulate proliferation of T cells. Unsurprising, neutralizing Abs to TGF-β and IL-10 showed a reverse effect.

## 2.7. Discussion

Five possibilities for tumor-associated DC may be involved in tumor escape from immunological attack have been reported. Firstly, the capability of phagocytosis and Ag processing of DC may be inhibited by tumors. Secondly tumors can inhibit maturation of tumor-infiltrating imDC, which in turn may induce T cell tolerance/dysfunction. Thirdly, tumors and/or the tumor microenvironment can secrete some chemokines such as MIP-3α, which may selectively chemoattract imDCs to tumor tissue, and may inhibit mature DCs into the tumor tissue. Fourth, the ability of DC migrating out of the tumor and into lymph nodes may be impaired in the tumor microenvironment [2,47–51]. Fifthly, tumors can educate DCs to differentiate into a regulatory DC subset, which contributes to the constitution of the immunosuppressive tumor microenvironment and promotes tumor immune escape [8].

In our research, we proved that tumor supernatants could disturb the development of DC, and induce myeloid precursor cells into CD11b^+^Gr1^+^ cells with immune suppressive function. We considered that these cells may have a possibility to be MDSC. It has been shown that regulatory DC could be induced by tumors via TGF-β and PGE_2_[8]. It is noteworthy that in this research, our MDSC were induced from myeloid precursor cells and regulatory DC were derived from immature dendritic cells.

We found downregulation of Twist and upregulation of miR-34a in tumor cell lines could reduce the ability of tumor supernatants to induce myeloid precursor into MDSC. TGF-β and IL-10 may be involved in the process.

Tumor cells secreted many immune suppressing cytokines such as TGF-β, VEGF, IL-10 and PGE_2_. It was shown that tumors can educate DCs to differentiate into regulatory DC subsets with immunosuppressive function via TGF-β, and PGE_2_. In our data, myeloid precursor cells can be induced into MDSC, not DC via TGF-β and IL-10. Previous data indicated that MDSC was induced by activation via the IL-1β/IL-6 pathway [8], and we imagine that the mechanism involved in our research were different from this. So, it appears that immunosuppressive cytokines play multiple roles in DC development and differentiation.

Twist plays varied roles in cancer initiation, progression and metastasis. More specifically, Twist can: override oncogene-induced cell senescence and apoptosis [52–54]; increase cancer cell resistance to chemotherapy [55]; enhance cancer stem cell (CSC) populations [56–58]; and facilitate cancer cell invasion and metastasis [28,31,59–63]. In our research, we demonstrated that Twist plays a very indirect role; Twist siRNA could suppress the secretion of IL-10 and TGF-β, which played important roles in induction of MDSC. So, it seems that Twist not only changes the biological behavior of tumor cells, but also induces immunosuppressive cytokines, which help tumor cells escaping from immune attack However, the precise mechanism still needs further investigation. MicroRNA miR-34 was identified as a p53 target [40,42], and recently the miR-34 family was found to directly link p53 and Wnt, revealing the tight connection between loss of tumor suppressor function and activation of oncogenic signaling. As loss of wt-p53 or hyperactivation of Wnt is critical in maintaining cancer stem cell properties and in establishing the metastatic program, these observations indicate a mechanism of miR-mediated quasi-sufficiency which connects tumor suppressor and oncogenic signaling pathways, supporting a continuum model of human cancer. Interestingly, our data added the third role of miR-34a, we found miR-34 played an indirect role in inducing MDSC via TGF-β and IL-10. Factors that induce MDSC expansion included cyclooxygenase-2 (COX2), prostaglandins [64–66], stem-cell factor (SCF) [64], macrophage colony-stimulating factor (M-CSF), IL-6 [67], GM-CSF and vascular endothelial growth factor (VEGF) [68]. Activation is necessary, and many factors are involved in activation, which include IFN-γ [69,70], ligands for Toll-like receptors (TLRs), IL-13 [71], IL-4 and transforming growth factor-β (TGF-β) [71]. Our data indicated that TGF-β maybe also played an important role in MDSC differentiation.

In conclusion, our data showed that tumor supernatants could induce myeloid precursor cells into MDSC, not DC. More importantly, we elucidated the role of Twist and miR-34a in the process. Our data provides two new molecular regulators which were involved in the accumulation of MDSC.

## 3. Experimental Section

### 3.1. Mice and Treatment

Male and female wild-type C57BL/6 mice, 5–6 weeks of age, were purchased from the Chinese Academy of Sciences (Shanghai, China). DO11.10 OVA323–339-specific TCR-transgenic mice with C57BL/6 background were obtained from The Jackson Laboratory. All mice were housed in a Specific Pathogen-Free (SPF) facility for all experiments. All animal experiments were undertaken in accordance with the National Institute of Health “Guide for the Care and Use of Laboratory Animals” (NIH Publication No. 85–23, National Academy Press, Washington DC, revised 1996), with the approval of the Laboratory Animal Center of the Second Military Medical University, Shanghai. All efforts were made to minimize the number of animals used as well as any suffering. The CT-26 colon cancer cell line, the Hepa1-6 hepatoma cell line, and the NIH3T3 fibroblast lines were all purchased from the American Type Culture Collection and maintained in RPMI 1640 complete medium (PAA Laboratories, GmbH, Pasching, Austria) supplemented with 10% FCS (PAA Laboratories, GmbH, Pasching, Austria).

### 3.2. Reagents

Recombinant mouse GM-CSF, IL-4, TGF-β, IL-10, neutralizing Abs to TGF-β and IL-10 and ELISA kit for murine IL-12, IL-6, TNF-α, IL-10, TGF-β were purchased from R & D Systems. Fluorescein-conjugated mAb to CD4, CD11b, CD80, CD86, Ia, CD40, CD11c, CCR7, Gr1, and isotype control were purchased from Santa Cruz Biotechnology. Fluorescein-conjugated mAbs to Gr1 were obtained from eBioscience. 7-Amnioactinomycin D (7-AAD), LPS were from Sigma-Aldrich (Sigma-Aldrich, St. Louis, MO, USA). miRNA miR-34a mimics, antagonists, and negative control miRNA mimic (NC mimic) were obtained from Dharmacon (Dharmacon, Chicago, IL, USA) [72].

### 3.3. Preparation of Tumor Supernatants Treated Myeloid Precursor Cells

Myeloid precursor cells were obtained from mice as described previously [8,9,45,46]. NIH3T3, Hepa1-6, and CT26 cells or these transfected cells were cultured in six-well plates (1 × 10^6^ cells/well), 12 h later they were washed with PBS 3 times. Fresh culture medium was then added to the wells. Twenty four hours later, supernatants were collected and co-cultured with myeloid precursor cells separately for 4 days. On day 6, cells in all groups were washed by PBS 3 times for further experiments.

### 3.4. Flow Cytometry

The phenotype of the cells and T cell response were analyzed by LSRII flow cytometry (BD Biosciences, Becton Dickinson, Franklin Lakes, NJ, USA) as described previously [8,44,45].

### 3.5. Tumor Model, Preparation of DC and Spleen Cells

The tumor model was constructed as previously indicated [8]. Tumor cells (1 × 10^6^/500 mL) were inoculated into C57BL/6 mice s.c.; 2 weeks later, these mice were euthanized for isolation of spleen. After depletion of red blood cells, spleen cells were doubled-labeled with Ab to Gr1 and Ab to CD11b for cytometry analysis. Bone marrow mononuclear cells were prepared from C57BL/6 mouse (5–6 weeks old) femur bone marrow suspensions by depletion of red cells and then were cultured at a density 2 × 10^6^ cells/mL in 6-well plates in RPMI 1640 medium supplemented with 10% FCS, 10 ng/mL of recombinant mouse granulocyte-monocyte colony-stimulating factor and 1 ng/mL of recombinant mouse IL-4. Nonadherent cells were gently washed out on day 4 of culture. At day 5, the dendritic proliferating clusters were collected.

### 3.6. Assay for Cytokines and NO

Tumor cells or NIH3T3 cells were seeded into six-well plates at a density of 1.0 × 10^6^/ml/well. After cells were adhered on plates, they were washed with PBS 3 times. The fresh medium was then added to the wells, and 24 h later, the cytokines were assayed with ELISA kits. NO production was assayed by measurement of the nitrite concentration with the Griess assay.

### 3.7. Assays for Ag-Specific CD4^+^ T Cell Response

As mDC could effectively prime proliferation of OVA-specific CD4^+^ T cells [8], so Ag-specific CD4^+^ T cell response were performed to test the function of dendritic cells. For assay of Ag-specific CD4^+^ T cell proliferation splenic CD4^+^ T cells from DO11.10 OVA323–339-specific TCR-transgenic mice were positively selected with anti-CD4-coated microbeads (Miltenyi Biotec, Miltenyi Biotec, Bergisch Gladbach, GmbH, Germany) by MACS and the co-cultured with DCs treated as indicated in the presence of OVA323–339 peptide at a ratio of 1:10 (DC:T) in round-bottom 96-well plates (1 × 10^5^ cells/200μ/well) for 5 days. Proliferation of T cells was analyzed by double staining with anti-CD4^+^ and 7-AAD^−^ cells were counted by FACS.

### 3.8. Assay for Percent of MDSC and DC *In Vivo*

The tumor bearing mice models were constructed by s.c. injection tumor cells (1 × 10^6^/500 mL) After 2 weeks, the spleens were isolated, and splenic single suspension cells were prepared, then they were stained with Ab-CD11c^+^ conjugated FITC, Ab-CD11b^+^ conjugated PE or Ab-Gr1^+^ conjugated FITC as standard protocol indicated.

### 3.9. Real-Time RT-PCR

The method of PCR was described by Yang *et al.* [28]. Mouse Twist RT-PCR forward primer is CGGGTCATGGCTAACGTG, and its RT-PCR reverse primer is CAGCTTGCCATCTTGGAGTC. miRNA-34a TaqMan assays were used to quantify levels of mature miRNA as described previously [73]. The primers of miR-34a were previously shown [40].

### 3.10. Twist Plasmids and siRNA Transfection

The method of transfection of Twist was described by Yang *et al.* [28]. The mouse Twist cDNA was achieved from Dr. Fabienne Perrin-Schmitt at Universite Louis Pasteur and subcloned into pBABE-puro vector (pBABE-puro-mTwist). pBABE-puro vector without mTwist subcloned as control.

Three siRNA-coding oligos against mouse Twist were designed and verified to be specific to Twist by Blast search against the mouse genome. Twist-siRNA1-targetting sequence is AAGCTGAGCA AGATTCAGACC; Twist-siRNA2-targetting sequence is AGCGGGTCATGGCTAACGTGC; Twist-siRNA2-targetting sequence is AGGTACATCGACTTCCTGTAC. We found Twist-siRNA1 showed the most potent effect, so we used Twist-siRNA1 in following experiments.

### 3.11. Transfection of miR-34 Mimics and Combined Transfection

Tumor cells were transfected 24 h after being seeded in 6-well plates. miRNA mimics (100 pmol) in 200 μL of serum-free, antibiotic-free, medium were mixed with 5 μL of Lipofectamine 2000 transfection reagent (Invitrogen, Carlsbad, CA, USA) dissolved in 200 μL of the same medium and allowed to stand at room temperature for 20 min. The resulting 400 μL transfection solutions were then added to each well containing 1.6 mL of medium. Six hours later, the cultures were replaced with 2 mL fresh medium supplemented with 10% FBS and antibiotics [74]. The combined transfection of Twist plasmid and miR-34a mimics required two steps; firstly, cells were transfected Twist plasmid as before, then 6 h later, miR-34a mimics were transfected as the previous method indicated.

### 3.12. Statistical Analysis

Comparisons between experimental groups and relevant controls were performed by Student’s *t* test or ANOVA. All the statistical analyses were performed with SPSS 16.0 (SPSS, Chicago, IL, USA, 2008) and *p* < 0.05 was considered statistically significant.

## 4. Conclusions

In summary, we confirmed that tumor supernatants could disturb the development of DC, and induce myeloid precursor cells into CD11b^+^ Gr1^+^ cells with immune suppressive function. We guessed that these cells may be MDSC. Interestingly, we found downregulation of Twist and upregulation of miR-34a in tumor cell lines could reduce the ability of tumor supernatants to induce myeloid precursor into MDSC. TGF-β and IL-10 may be involved in the process.
